# Carbohydrates, proteins, fats and other essential components of food from native trees in West Africa

**DOI:** 10.1016/j.heliyon.2019.e01744

**Published:** 2019-05-22

**Authors:** Anne Mette Lykke, Elie Antoine Padonou

**Affiliations:** a Department of Bioscience, Aarhus University, Vejlsøvej 25, DK 8600, Silkeborg, Denmark; b School of Tropical Forestry, National University of Agriculture, BP 43, Kétou, Benin; c Faculty of Agronomic Sciences, University of Abomey-Calavi, Laboratory of Applied Ecology, 01 BP 526, Cotonou, Benin

**Keywords:** Agriculture, Food science, Food analysis, Nutrition

## Abstract

Native tree foods contribute to food and nutrition security, health and income generation in sub-Saharan Africa. However, the specific contribution of native tree foods to nutrition is poorly documented in science and often not acknowledged in poverty reduction strategies. This review gives an overview on the content of carbohydrates, proteins, fat, fibers, ash and dry matter of 98 native food tree species from sub-Saharan Africa. Data were grouped according to the food providing organ (seeds, fruits and leaves). In general, seeds had high content of fat, protein and dry matter; while leaves had high content of protein and ash. There was no significant difference between the three organs on the content of fibers and carbohydrate. Some tree foods species were good sources to provide carbohydrates, proteins, fat, fibers, ash and dry matter.

## 1 Introduction

In sub-Saharan Africa, it is well-known that indigenous trees traditionally contribute to food and nutrition security, health and income generation (Hyacinthe et al*.,* 2015; Otori and Mann, 2014; Stadlmayr et al., 2013). The more specific contribution of food from native trees to nutrition, however, is poorly documented in science and often not acknowledged in poverty reduction strategies (Schreckenberg et al*.,* 2006; Ngome et al., 2017). Therefore, several trees may be considered for food uses, but their nutritional value is underestimated (FAO, 2013). Information on the nutrient composition of food is essential to estimate adequate nutrient intake both at individual and group levels (Joyanes and Lema, 2006). This information may facilitate the selection of priority tree food species for domestication programs aimed at improving food and nutrition security and income generation (Stadlmayr et al., 2013) as well as for natural resource management and conservation.

Nutritional components generally analysed are carbohydrates, proteins, fat, fibers, ash, vitamins, minerals and dry matter. Carbohydrates hold a special place in human nutrition providing the largest single source of energy in the diet and satisfying instinctual desire for sweetness (Brand-Miller, 2002). A high carbohydrate content is a major source of readily available energy (Assogbadjo et al., 2012; Bamidele et al., 2015). Proteins are fundamental elements for metabolism of enzymes, hormones and many other molecules essential for life. Proteins are composed of 20 amino acids of which nine are essential and need to be provided through the diet (histidine, isoleucine, leucine, lysine, methionine, phenylalanine, threonine, tryptophan and valine). In early childhood a number of amino acids, which are not essential in adults, cannot be formed in adequate amounts. These are conditionally essential, because of the limited ability of their endogenous formation relative to the magnitude of demand (arginine, cysteine, glycine, glutamine, histidine, proline and tyrosine) (Jackson, 2002). There may be disease situations during adult life whereby a particular amino acid, or group of amino acids, becomes conditionally essential (Jackson, 2002). Some tree food species are promising as sources of dietary protein and amino acid supplement for domestic and industrial use (Djenontin et al., 2009; Igwenyi and Akubugwo, 2010; Lohlum, 2010; Ayessou et al., 2014; Otori and Mann, 2014). Fats are a major source of energy and are the essential fuel for the brain and growing fetus (Stubbs et al., 2018). They enhance flavour and palatability of food and make an important contribution to health containing essential fatty acids that cannot be synthesized in the body and are furthermore required for a range of metabolic and physiological processes to maintain the structural and functional integrity of cell membranes (Mann and Skeaff, 2002). High content of fat make certain oils from native trees a good alternative or supplement to conventional oil (Bazongo et al., 2014; Niyi, 2014). Fiber has many health benefits and can reduce diabetes (Bazongo et al., 2014) and high blood cholesterol (Liu et al., 2000). Ash content is obtained by burning away of organic materials and gives a measure of the contents inorganic minerals (Adubiaro et al., 2011; Amoo et al., 2012; Ayessou et al., 2014). High content of dry matter in food is an advantage for high shelve-life because it prevents microbial spoilage and pest attack during storage (Magaia et al., 2013; Honfo et al., 2014).

The present review gives an overview of the content of carbohydrates, proteins, fat, fibers, ash and dry matter of 98 native trees food species from West Africa based on scientific literature.

## 2 Methodology

The review was based on literature on tree food species from West Africa. Species were selected based on floras, plant lists and books (Akoègninou et al., 2006; Arbonnier, 2002; Matig et al., 2006; Codjia et al., 2015; Awodoyin et al., 2015) with the following search terms: “proximate composition”, “carbohydrate”, “protein”, “fat”, “lipid”, “fiber”, “ash” and “dry matter” as these are the components of proximate composition. For each variable considered, the reported values were converted into the same units and synthetized in tables.

Data were grouped according to the food providing organ [seeds, fruit (all other fruit parts besides of the seeds) and leaves] and analyzed using ANOVA, Student-Newman-Keuls Test and Principal Component Analysis (PCA) based on correlation. No data transformation was applied, as data met the assumption of normality and homoscedasticity based on Ryan-Joiner test of normality and the Levene test for homogeneity of variances. All the analyses were performed using R statistical software (R Core Team, 2013).

## 3 Results

### 3.1 Proximate composition of the organs

Seeds, fruit and leaves had significantly different proximate composition (Appendix 1). The highest protein content was found in seeds and leaves, highest fat content in seeds, highest ash content in leaves and highest dry matter content in seeds. There was no significant difference between the three organs concerning the content of carbohydrate and fibers.

### 3.2 Seeds

**Fig. 1:**
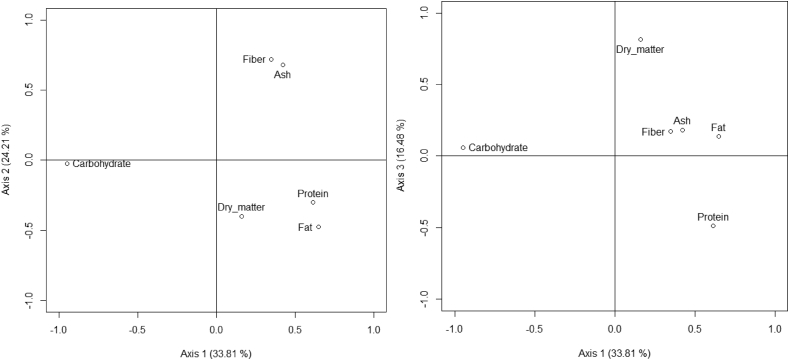
Relation of the specific components of seeds based on PCA analysis (axis 1 vs axis 2 and axis 1 vs axis 3).

**Fig. 2:**
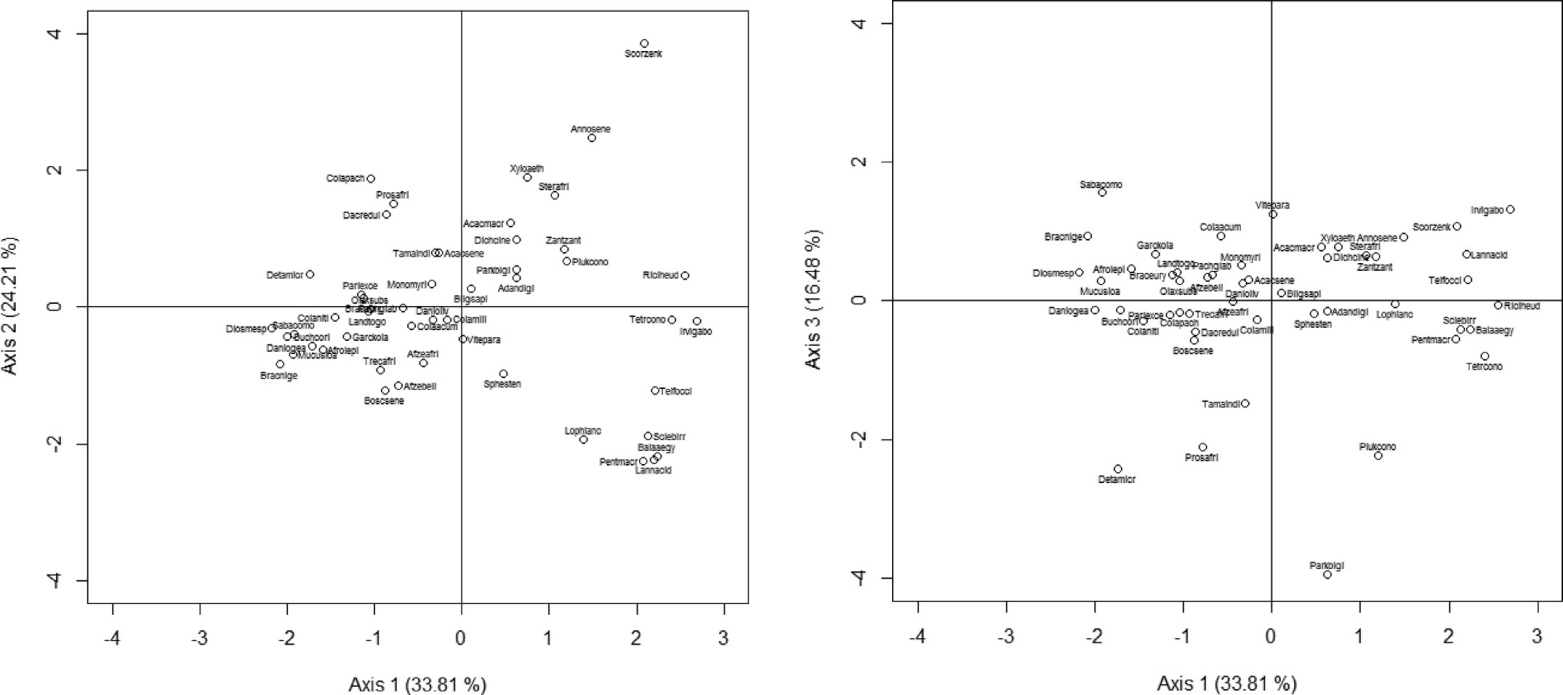
Species distribution of the proximate composition of the seeds based on PCA (axis 1 vs axis 2 and axis 1 vs axis 3). Acacmacr: *Acacia macrostachya*, Acacsene: *Acacia senegal*, Adandigi: *Adansonia digitata*, Afrolepi: *Afrostyrax lepidophyllus*, Afzeafri: *Afzelia africana*, Afzebell: *Afzelia bella*, Annosene: *Annona senegalensis*, Balaaegy: *Balanites aegyptiaca*, Bligsapi: *Blighia sapida*, Boscsene: *Boscia senegalensis*, Braceury: *Brachystegia eurycoma*, Bracnige: *Brachystegia nigerica*, Buchcori: *Buchholzia coriacea*, Colaacum: *Cola acuminata*, Colamill: *Cola millenii*, Colaniti: *Cola nitida*, Colapach: *Cola pachycarpa*, Dacredul: *Dacryodes edulis*, Daniogea: *Daniellia ogea*, Danioliv: *Daniellia oliveri*, Detamicr: *Detarium microcarpum*, Dichcine: *Dichrostachys cinerea*, Diosmesp: *Diospyros mespiliformis*, Garckola: *Garcinia kola*, Irvigabo: *Irvingia gabonensis*, Landtogo: *Landolphia togolana*, Lannacid: *Lannea acida*, Lophlanc: *Lophira lanceolata*, Monomyri: *Monodora myristica*, Mucusloa: *Mucuna sloanei*, Olaxsubs: *Olax subscorpioides*, Pachglab: *Pachira glabra*, Parkbigl: *Parkia biglobosa*, Pentmacr: *Pentaclethra macrophylla*, Pariexce: *Parinari excelsa*, Prosafri: *Prosopis africana*, Riciheud: *Ricinodendron heudelotii*, Sabacomo: *Saba comorensis*, Sclebirr: *Sclerocarya birrea*, Scorzenk: *Scorodophloeus zenkeri*, Sphesten: *Sphenostylis stenocarpa*, Sterafri: *Sterculia africana*, Tamaindi: *Tamarindus indica*, Telfocci: *Telfairia occidentalis*, Plukcono: *Plukenetia conophora*, Trecafri: *Treculia africana*, Tetrcono: *Tetracarpidium conophorum*, Vitepara: *Vitellaria paradoxa*, Xyloaeth: *Xylopia aethiopica*, Zantzant: *Zanthoxylum zanthoxyloides*.

The PCA performed on the proximate composition of seeds revealed that the first three axes explained 77% of the variation in proximate composition (Fig. 1). Fat (71%) and protein (56%) were positively correlated with axis 1, while carbohydrate (-95%) was negatively correlated with this axis. The group of species with high content of fat were *Irvingia gabonensis, Telfairia occidentalis, Sclerocarya birrea, Lophira lanceolata, Balanites aegyptiaca, Pentaclethra macrophylla, Vitellaria paradoxa, Cola millenii. Ricinodendron heudelotii* and *Annona senegalensis* (Appendix 2, Fig. 2). The species with highest content in protein were *Tetracarpidium conophorum*, *Balanites aegyptiaca*, *Ricinodendron heudelotii*, *Pentaclethra macrophylla*, *Sphenostylis stenocarpa*, *Parkia biglobosa*, *Sclerocarya birrea*, *Lophira lanceolata*, *Sterculia africana* and *Boscia senegalensis* (Appendix 2, Fig. 2). The species with high content of carbohydrate were *Brachystegia nigerica*, *Diospyros mespiliformis*, *Saba comorensis*, *Mucuna sloanei*, *Daniellia ogea*, *Cola pachycarpa*, *Afrostyrax lepidophyllus*, *Buchholzia coriacea*, *Detarium microcarpum* and *Olax subscorpioides* (Appendix 2, Fig. 2).

Ash (72%) and fibers (75%) were positively correlated with axis 2 (Fig. 1). The species with high content of ash were *Annona senegalensis*, *Cola pachycarpa*, *Ricinodendron heudelotii*, *Scorodophloeus zenkeri*, *Dichrostachys cinerea*, *Zanthoxylum zanthoxyloides*, *Irvingia gabonensis*, *Xylopia aethiopica*, *Tetracarpidium conophorum* and *Parinari excelsa* (Appendix 2, Fig. 2). The species with highest content of fiber were *Scorodophloeus zenkeri*, *Sterculia africana*, *Acacia macrostachya*, *Acacia senegal*, *Xylopia aethiopica*, *Dacryodes edulis*, *Annona senegalensis*, *Blighia sapida*, *Adansonia digitata* and *Monodora myristica* (Appendix 2, Fig. 2).

Dry matter (90.86%) was positively correlated with axis 3. The species with high content of dry matter were *Cola acuminata*, *Saba comorensis*, *Sphenostylis stenocarpa*, *Tetracarpidium conophorum*, *Brachystegia nigerica*, *Balanites aegyptiaca*, *Vitellaria paradoxa*, *Pentaclethra macrophylla*, *Sterculia africana* and *Irvingia gabonensis* (Appendix 2, Fig. 2).

### 3.3 Fruit

**Fig. 3:**
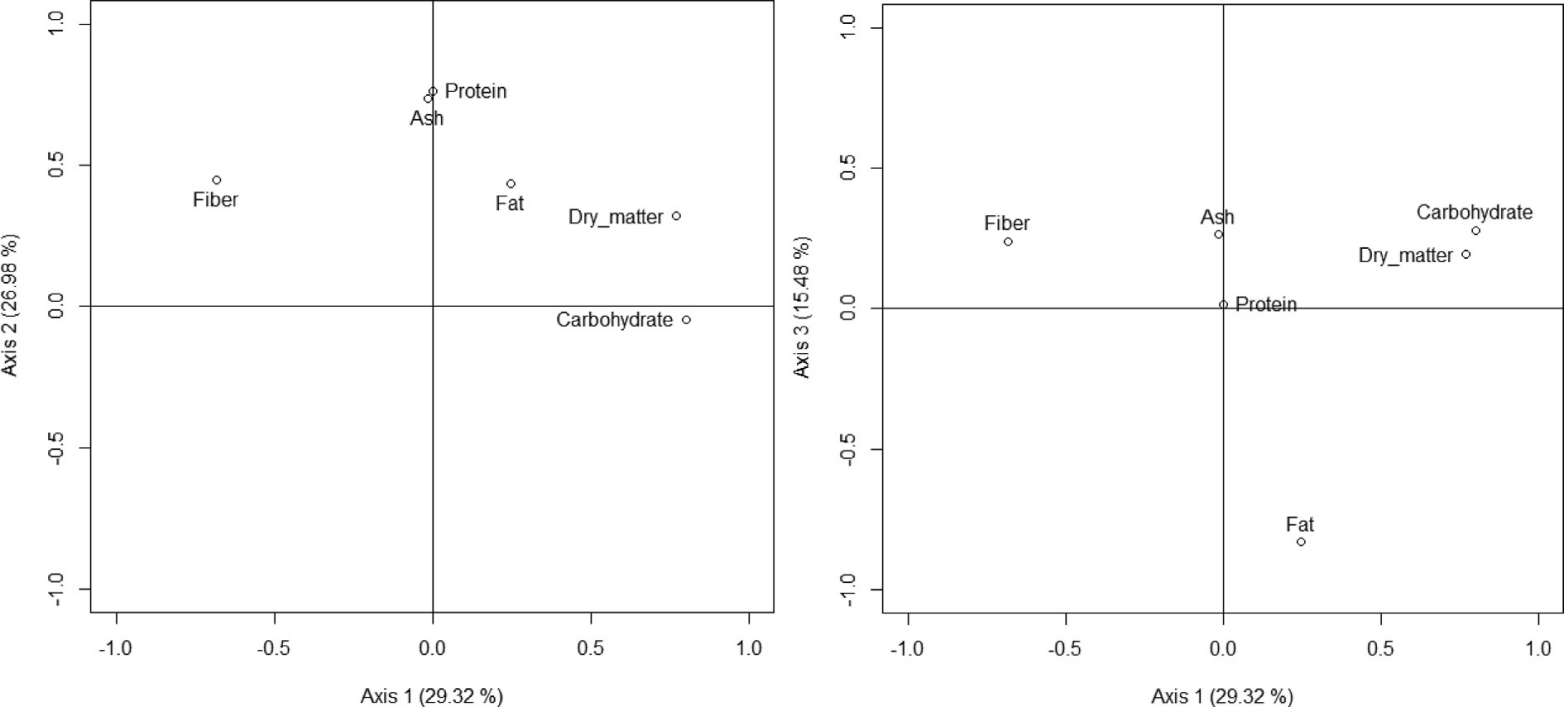
Relation of the nutrition components of fruit based on PCA analysis (axis 1 vs axis 2 and axis 1 vs axis 3).

**Fig. 4:**
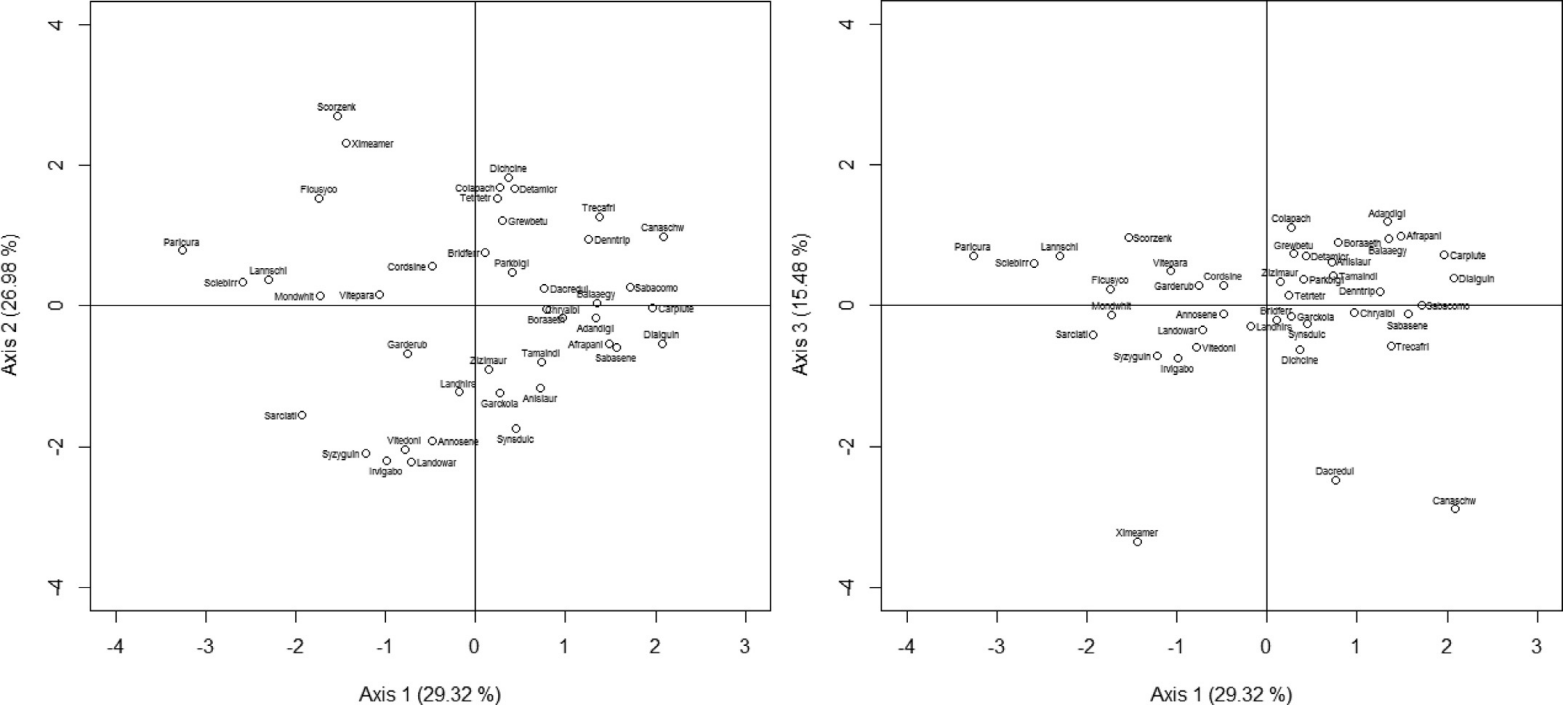
Species distribution of the proximate composition of fruit based on PCA (axis 1 vs axis 2 and axis 1 vs axis 3). Adandigi: *Adansonia digitata*, Afrapani: *Afraegle paniculata*, Anislaur: *Anisophyllea laurina*, Annosene: *Annona senegalensis*, Balaaegy: *Balanites aegyptiaca*, Boraaeth: *Borassus aethiopum*, Bridferr: *Bridelia ferruginea*, Canaschw: *Canarium schweinfurthii*, Carplute: *Carpolobia lutea*, Chryalbi: *Chrysophyllum albidum*, Colapach: *Cola pachycarpa*, Cordsine: *Cordia sinensis*, Dacredul: *Dacryodes edulis*, Denntrip: *Dennettia tripetala*, Detamicr: *Detarium microcarpum*, Dialguin: *Dialium guineense*, Ficusyco: *Ficus sycomorus*, Garckola: *Garcinia kola*, Garderub, *Gardenia erubescens*, Grewbetu: *Grewia betulaefolia*, Irvigabo: *Irvingia gabonensis*, Landhirs*: Landolphia hirsuta*, Landowar: *Landolphia owariensis*, Lannschi: *Lannea schimperi*, Mondwhit: *Mondia whitei*, Paricura: *Parinari curatellifolia*, Parkbigl: *Parkia biglobosa*, Sabacomo: *Saba comorensis*, Sabasene: *Saba senegalensis*, Sarclati: *Sarcocephalus latifolius*, Sclebirr: *Sclerocarya birrea*, Scorzenk: *Scorodophloeus zenkeri*, Synsdulc: *Synsepalum dulcificum*, Syzyguin: *Syzygium guineense*, Tamaindi: *Tamarindus indica*, Tetrtetr: *Tetrapleura tetraptera*, Trecafri: *Treculia africana*, Vitepara: *Vitellaria paradoxa*, Vitedoni: *Vitex doniana*, Ximeamer: *Ximenia americana*, Zizimaur: *Ziziphus mauritiana*.

A positive correlation was found between dry matter (83%), carbohydrate (82%) and axis 1 (Fig. 3). Fibers (-62%) was negatively correlated with axis 1. Species with high content of dry matter were *Saba comorensis*, *Saba senegalensis*, *Dialium guineense*, *Detarium microcarpum*, *Afraegle paniculata*, *Borassus aethiopum*, *Bridelia ferruginea*, *Dennettia tripetala*, *Adansonia digitata* and *Canarium schweinfurthii* (Appendix 3, Fig. 4). The Species with high content of carbohydrate were *Anisophyllea laurina*, *Carpolobia lutea*, *Dialium guineense*, *Adansonia digitata*, *Afraegle paniculata*, *Saba senegalensis*, *Saba comorensis*, *Chrysophyllum albidum*, *Balanites aegyptiaca* and *Cola pachycarpa* (Appendix 3, Fig. 4). Species with high content of fibers were *Parinari curatellifolia*, *Lannea schimperi*, *Sclerocarya birrea*, *Ficus sycomorus*, *Bridelia ferruginea*, *Ximenia Americana*, *Vitellaria paradoxa*, *Gardenia erubescens*, *Detarium microcarpum* and *Borassus aethiopum* (Appendix 3, Fig. 4).

Protein (74.92%) and ash (76.77%) were positively correlated with axis 2 (Fig. 3). The species with high content of protein were *Detarium microcarpum*, *Treculia africana*, *Ximenia americana*, *Cordia sinensis*, *Grewia betulaefolia*, *Dennettia tripetala*, *Carpolobia lutea*, *Cola pachycarpa*, *Tetrapleura tetraptera* and *Dacryodes edulis* (Appendix 3, Fig. 4). The species with high content of ash were *Cola pachycarpa*, *Ficus sycomorus*, *Tetrapleura tetraptera*, *Saba comorensis*, *Mondia whitei*, *Sclerocarya birrea*, *Grewia betulaefolia*, *Parkia biglobosa*, *Canarium schweinfurthii* and *Anisophyllea laurina* (Appendix 3, Fig. 4).

Fat (-84.83%) was negatively correlated with axis 3 (Fig. 3). The species with high content of fat were *Canarium schweinfurthii*, *Ximenia americana*, *Dacryodes edulis*, *Saba comorensis*, *Treculia africana*, *Saba senegalensis*, *Bridelia ferruginea*, *Tetrapleura tetraptera*, *Ficus sycomorus* and *Chrysophyllum albidum* (Appendix 3, Fig. 4).

### 3.4 Leaves

**Fig. 5:**
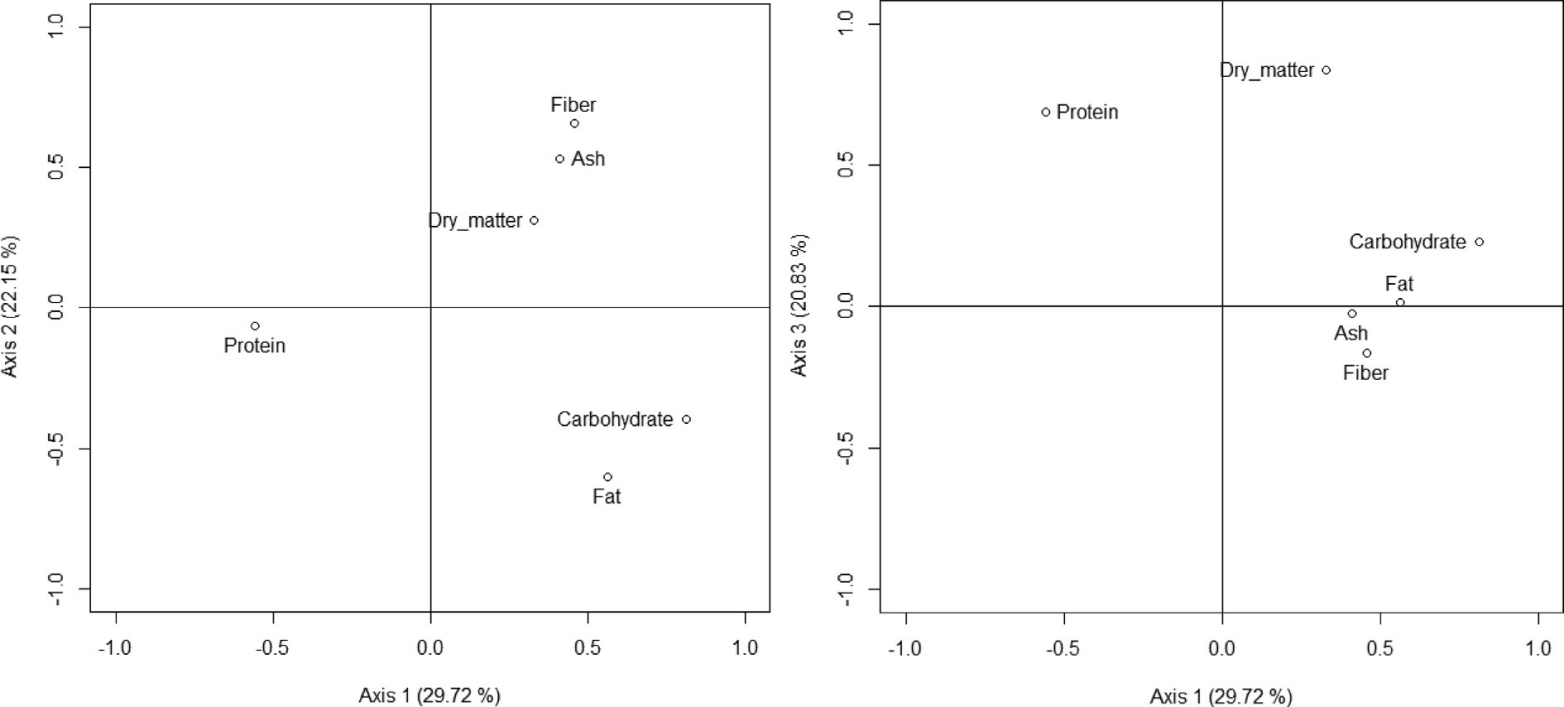
Relation of the specific components of leaves based on PCA analysis (axis 1 vs axis 2 and axis 1 vs axis 3).

**Fig. 6:**
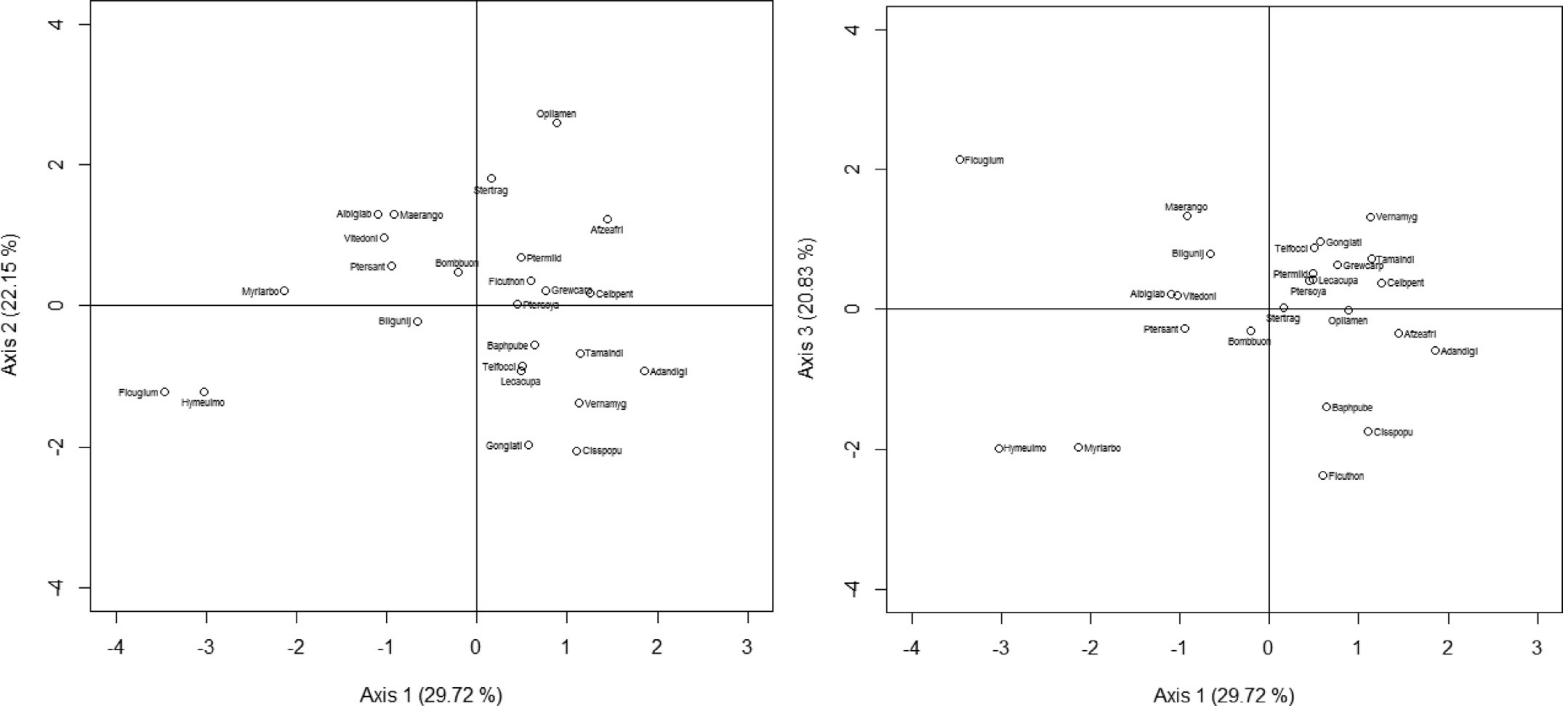
Species distribution of the proximate composition of leaves based on PCA (axis 1 vs axis 2 and axis 1 vs axis 3). Adandigi: *Adansonia digitata*, Afzeafri: *Afzelia africana*, Albiglab: *Albizia glaberrima*, Baphpube: *Baphia pubescens*, Bligunij: *Blighia unijugata*, Bombbuon: *Bombax buonopozense*, Ceibpent: *Ceiba pentandra*, Cisspopu: *Cissus populnea*, Ficuglum: *Ficus glumosa*, Ficuthon: *Ficus thonningii*, Gonglati: *Gongronema latifolium*, Grewcarp: *Grewia carpinifolia*, Hymeulmo: *Hymenocardia ulmoides*, Lecacupa: *Lecaniodiscus cupanioides*, Maerango: *Maerua angolensis*, Myriarbo: *Myrianthus arboreus*, Opilamen: *Opilia amentacea*, Ptermild: *Pterocarpus mildbraedii*, Ptersant: *Pterocarpus santalinoides*, Ptersoya: *Pterocarpus soyauxii*, Stertrag: *Sterculia tragacantha*, Tamaindi: *Tamarindus indica*, Telfocci: *Telfairia occidentalis*, Vernamyg: *Vernonia amygdalina*, Vitedoni: *Vitex doniana*.

Leave content of carbohydrate (77%), fat (58%), fibers (56%) and ash (56%) were positively correlated with axis 1 (Fig. 5). The species with high content of carbohydrate were *Tamarindus indica*, *Vernonia amygdalina*, *Adansonia digitata*, *Cissus populnea*, *Telfairia occidentalis*, *Lecaniodiscus cupanioides*, *Gongronema latifolium*, *Grewia carpinifolia*, *Pterocarpus soyauxii* and *Baphia pubescens* (Appendix 4, Fig. 6). The species with high content of fat were *Cissus populnea*, *Gongronema latifolium*, *Ceiba pentandra*, *Vernonia amygdalina*, *Adansonia digitata*, *Ficus thonningii*, *Baphia pubescens*, *Pterocarpus mildbraedii*, *Telfairia occidentalis* and *Afzelia africana* (Appendix 4, Fig. 6). The species with high content of fibers were *Afzelia africana*, *Ficus thonningii*, *Sterculia tragacantha*, *Ceiba pentandra*, *Albizia glaberrima*, *Pterocarpus santalinoides*, *Vitex doniana*, *Pterocarpus mildbraedii*, *Grewia carpinifolia* and *Opilia amentacea* (Appendix 4, Fig. 6). The species with high content of ash were *Opilia amentacea*, *Pterocarpus mildbraedii*, *Maerua angolensis*, *Baphia pubescens*, *Sterculia tragacantha*, *Adansonia digitata*, *Cissus populnea*, *Ceiba pentandra*, *Grewia carpinifolia* and *Ficus thonningii* (Appendix 4, Fig. 6).

Dry matter (67.38%) was positively correlated with axis 2. The species with high content of dry matter were *Tamarindus indica*, *Ceiba pentandra*, *Grewia carpinifolia*, *Maerua angolensis*, *Vernonia amygdalina*, *Opilia amentacea*, *Afzelia africana*, *Telfairia occidentalis*, *Pterocarpus soyauxii* and *Lecaniodiscus cupanioides* (Appendix 4, Fig. 6).

Protein (58.32%) was positively correlated with axis 3 (Fig. 5). The species with high content of protein were *Ficus glumosa*, *Maerua angolensis*, *Vitex doniana*, *Albizia glaberrima*, *Vernonia amygdalina*, *Pterocarpus santalinoides*, *Blighia unijugata*, *Gongronema latifolium*, *Pterocarpus mildbraedii* and *Telfairia occidentalis* (Appendix 4, Fig. 6).

## 4 Conclusion

A large number of native species from West Africa have good potentials for food and nutritional supplements. Carbohydrates and fibers are found in equal amounts in seeds, fruits and leaves. Seeds are important sources for both protein and fat. Leaves are important sources for protein and minerals. Fruits generally have lower content of protein, fat and minerals, but these components are still present.

Species with high amounts of protein and fat in seeds and fruits are often low in carbohydrates. This is in contrast to leaves, where the species with highest carbohydrate and fat content often are low in protein. It is therefore important to focus more specifically on the qualities of each food source in order to meet the particular local nutritional needs.

The species reviewed in this study are often directly available to local communities, however they are often neglected in poverty and nutritional strategies. For instance *Vitellaria paradoxa* is well known for shea butter production, but several species with higher fat content, such as *Irvingia gabonensis, Sclerocarya birrea* and *Balanites aegyptiaca*, are rarely use, and could have high potentials for improving food and nutritional security. Only few of the species are widely known and exported, but these few species have large economic potentials, eg *Vitallaria paradoxa* and *Elaeis guineensis*. Many other species have similar properties, which indicates a large unexploited potential from native species in West Africa.

For many species only few chemical analyses have been published and some of the published data have been found doubtful during the verification of data for the review. Therefore further analyses are needed to verify the reported nutritional content and to test for substances that could have toxic or pathogenic longterm effects that have not been noticed based on the traditional uses.

Desertification and forest destruction in West Africa is likely to eliminate nutritionally and economically valuable native species to give place for less valuable crops, only because the value was not been realized outside particular communities. Greater sustainable use of seeds, fruits and leaves by local communities could go hand in hand with nature conservation.

## Declarations

### Author contribution statement

All authors listed have significantly contributed to the development and the writing of this article.

### Funding statement

This work was supported by Agropolis, Cariplo and Daniel & Nina Carasso foundations via the TREEFOOD project.

### Competing interest statement

The authors declare no conflict of interest.

### Additional information

No additional information is available for this paper.
