# Supplementary material for: Carbohydrates, proteins, fats and other essential components of food from native trees in West Africa
Source: Heliyon. 2019 May 22;5(5):e01744. doi: 10.1016/j.heliyon.2019.e01744 (PMC6531672; doi:10.1016/j.heliyon.2019.e01744)
Supplement: Appendix 1 [file mmc1.docx]

Appendix 1. Average proximate composition of seeds, fruits and leaves from native trees

| Source | Carbohydrate  (%) | Protein  (%) | Fat  (%) | Fiber  (%) | Ash  (%) | Dry matter  (%) |
| --- | --- | --- | --- | --- | --- | --- |
| Seeds | 42.94a | 18.44a | 17.60a | 13.95a | 4.12b | 85.76a |
| Fruits | 45.10a | 6.04b | 6.50b | 13.28a | 4.86b | 58.37b |
| Leaves | 40.40a | 18.40a | 4.80b | 17.76a | 8.10a | 67.21b |
| ANOVA  F value | 0.61ns | 5.42*** | 23.06*** | 1.28ns | 31.00*** | 21.81*** |

ns, non-significant at P 0.05; ***, significant at P ≤ 0.001. The values with same letter are not significantly different based on Student-Newman-Keuls Test.
