# Supplementary material for: Carbohydrates, proteins, fats and other essential components of food from native trees in West Africa
Source: Heliyon. 2019 May 22;5(5):e01744. doi: 10.1016/j.heliyon.2019.e01744 (PMC6531672; doi:10.1016/j.heliyon.2019.e01744)
Supplement: Appendix 2 [file mmc2.docx]

Appendix 2. Average values of proximate composition in seeds

| Species | Fat | Protein | Ash | Fiber | Carbohydrate | Dry matter | Authors |
| --- | --- | --- | --- | --- | --- | --- | --- |
| *Acacia macrostachya* DC. | 8.96 | 12.16 | 4.26 | 22.70 | 24.40 | 93.37 | Savadogo et al. 2011 |
| *Acacia senegal.* (L.) Willd. | 3.30 | 20.60 | 2.60 | 22.70 | 51.70 | 93.37 | Kyari et al. 2017 |
| *Adansonia digitata* L. | 22.53 | 23.80 | 5.85 | 14.18 | 44.73 | 88.77 | Ajayi 2009; Assogbadjo et al. 2012; Ezeagu 2017; Greene 1932; Nnam and Obiakor 2003; Lockett et al. 2000; Magaia et al. 2013; Murray et al. 2001; Nkafamiya et al. 2007; Osman 2004; Parkouda et al. 2012; Emmanuel et al. 2011 |
| *Afrostyrax lepidophyllus* Mildbr. | 13.20 | 7.70 | 3.00 | 4.40 | 68.30 | 92.20 | Ene-Obong et al. 2018 |
| *Afzelia africana* Pers. | 15.60 | 18.60 | 3.39 | 4.59 | 47.40 | 92.34 | Ajah and Madubuike 1997; Dike 2010; Igwenyi and Azoro 2014; Igwenyi and Akubugwo 2010; Njoku et al. 1999; Niyi 2014 ; Ogunlade et al. 2011; Onweluzo and Morakinyo 1997 |
| *Afzelia bella* Harms | 23.17 | 13.09 | 2.97 | 2.95 | 53.96 | 93.19 | Ezeagu et al. 2000 |
| *Annona senegalensis* Pers. | 24.00 | 8.80 | 12.10 | 17.60 | 25.30 | 87.80 | Yisa et al. 2010 |
| *Balanites aegyptiaca* (L.) Delile | 41.50 | 40.91 | 2.71 | 5.65 | 11.80 | 96.91 | Elbadawi et al. 2017 |
| *Blighia sapida* K.D.Koenig | 6.60 | 22.40 | 3.50 | 15.60 | 39.45 | 93.30 | Djenontin et al. 2009 |
| *Boscia senegalensis* Lam. | 1.11 | 24.23 | 1.16 | 3.00 | 45.29 | 92.54 | Edwige et al. 2014 |
| *Brachystegia eurycoma* Harms | 8.75 | 9.38 | 3.65 | 9.95 | 58.99 | 90.90 | Ajah and Madubuike 1997; Bolanle et al. 2014 |
| *Brachystegia nigerica* Hoyle & A.P.D.Jones | 6.42 | 7.45 | 3.07 | 2.30 | 78.12 | 97.35 | Igwenyi and Azoro 2014; Igwenyi and Akubugwo 2010 |
| *Buchholzia coriacea* Engl. | 4.03 | 12.92 | 2.66 | 3.54 | 66.91 | 89.88 | Ijarotimi et al. 2015 |
| *Cola acuminata* (P.Beauv.) Schott & Endl. | 1.20 | 10.64 | 3.50 | 7.35 | 34.56 | 98.80 | Dah-Nouvlessounon et al. 2015 |
| *Cola millenii* K.Schum. | 28.57 | 9.72 | 4.44 | 6.44 | 41.30 | 84.00 | Bello et al. 2008; Ojelere 2014 |
| *Cola nitida* (Vent.) Schott & Endl. | 0.20 | 10.06 | 3.00 | 4.31 | 51.54 | 87.53 | Dah-Nouvlessounon et al. 2015 |
| *Cola pachycarpa* K.Schum. | 0.69 | 11.29 | 10.78 | 6.20 | 70.94 | 82.85 | Essein et al. 2017 |
| *Dacryodes edulis* (G.Don) H.J.Lam | 11.97 | 5.90 | 3.56 | 18.59 | 53.06 | 79.48 | Akanni et al. 2005; Ajayi 2009; Ebana et al. 2017 |
| *Daniellia ogea* (Harms) Holland | 0.46 | 13.49 | 1.87 | 6.35 | 74.32 | 90.14 | Ezeagu et al. 2000 |
| *Daniellia oliveri* Hutch. & Dalziel | 7.71 | 22.15 | 5.18 | 7.28 | 52.50 | 94.92 | Adubiaro et al. 2011; Otori and Mann 2014 |
| *Detarium microcarpum* Guill. & Perr. | 6.19 | 14.94 | 3.06 | 5.14 | 65.51 | 68.25 | Akpata and Miachi 2001; Dike 2010; Eromosele et al. 1994; Igwenyi and Azoro 2014; Igwenyi and Akubugwo 2010; Lockett et al. 2000; Njoku et al. 1999 |
| *Dichrostachys cinerea* (L.) Wight & Arn. | 11.20 | 8.60 | 9.80 | 4.00 | 17.80 | 91.00 | Bouba et al. 2012 |
| *Diospyros mespiliformis* Hochst. ex A.DC. | 5.46 | 5.46 | 2.89 | 5.00 | 77.21 | 91.01 | Ezeagu et al. 2000 |
| *Garcinia kola* Heckel | 8.70 | 7.88 | 2.75 | 6.98 | 57.94 | 94.43 | Dah-Nouvlessounon et al. 2015; Ebana et al. 2017 |
| *Irvingia gabonensis* (Aubry-Lecomte ex O'Rorke) Baill. | 60.97 | 13.54 | 8.91 | 9.97 | 7.99 | 95.11 | Bamidele et al. 2015; Dosumu et al. 2012; Akanni et al. 2005 |
| *Landolphia togolana* (Hallier f.) Pichon | 9.90 | 10.81 | 4.25 | 7.38 | 59.62 | 91.96 | Akoja and Amoo 2011 |
| *Lannea acida* A.Rich. | 64.9 | 21.14 | 3.11 | 5.4 | 10.85 | 96.76 | Bazongo et al. (2014) |
| *Lophira lanceolata* Tiegh. ex Keay | 44.31 | 28.45 | 1.45 | 8.43 | 24.05 | 94.86 | Eromosele and Eromosele 1993; Lohlum 2010 |
| *Monodora myristica* (Gaertn.) Dunal | 14.59 | 14.13 | 4.66 | 13.27 | 52.83 | 92.4 | Ajayi 2009; Dike 2010; Ene-Obong et al. 2016 |
| *Mucuna sloanei* Fawc. & Rendle | 4.85 | 11.65 | 2.67 | 3.55 | 75.3 | 93.21 | Igwenyi and Azoro 2014; Igwenyi and Akubugwo 2010; Akanni et al. 2005 |
| *Olax subscorpioides* Oliv. | 3.66 | 16.21 | 4.56 | 7.27 | 61.70 | 93.38 | Otori and Mann 2014 |
| *Pachira glabra* Pasq. | 15.29 | 10.38 | 4.34 | 8.56 | 52.32 | 90.87 | Ogunlade et al. 2011 |
| *Parkia biglobosa* (Jacq.) G.Don | 18.46 | 33.43 | 3.94 | 7.72 | 23.85 | 60.37 | Alabi et al. 2005; Dosumu et al. 2012; Lockett et al. 2000; Ogunyinka et al. 2017 |
| *Pentaclethra macrophylla* Benth. | 38.58 | 37.59 | 1.78 | 4.66 | 4.25 | 95.52 | Ajah and Madubuike 1997; Ajayi 2009; Balogun 2013 |
| *Parinari excelsa* Sabine | 6.95 | 11.76 | 5.91 | 2.69 | 59.38 | 86.69 | Ojelere 2014 |
| *Prosopis africana* (Guill. & Perr.) Taub. | 6.81 | 16.92 | 5.54 | 11.69 | 54.31 | 69.05 | Achi et al. 2004; Barminas et al. 1998; Dosumu et al. 2012; Lockett et al. 2000 |
| *Ricinodendron heudelotii* (Baill.) Heckel | 24.50 | 40.45 | 9.95 | 11.70 | 19.25 | 94.85 | Ene-Obong et al. 2016; Clergé Tchiegang et al. 2006 |
| *Saba comorensis* (Bojer ex A.DC.) Pichon | 11.90 | 0.32 | 3.95 | 5.50 | 76.39 | 98.50 | Omale et al. 2010 |
| *Sclerocarya birrea* (A.Rich.) Hochst. | 50.44 | 30.89 | 4.21 | 2.51 | 10.15 | 91.70 | Eromosele and Eromosele 1993; Muhammad et al. 2011 |
| *Scorodophloeus zenkeri* Harms | 2.00 | 12.20 | 9.90 | 35.00 | 1.50 | 90.50 | Bouba et al. 2012 |
| *Sphenostylis stenocarpa* (A.Rich.) Harms | 9.49 | 37.21 | 5.35 | 3.55 | 44.40 | 98.04 | Chinedu and Nwinyi 2012 |
| *Sterculia africana* (Lour.) Fiori | 6.84 | 24.90 | 5.69 | 27.55 | 34.82 | 95.36 | Emmanuel et al. 2011 |
| *Tamarindus indica* L. | 8.31 | 19.99 | 4.53 | 11.01 | 42.44 | 77.29 | Ajayi et al. 2006; Ishola et al. 1990; Lockett et al. 2000; Pugalenthi et al. 2004; Siddhuraju et al. 1995; Yusuf et al. 2007 |
| *Telfairia occidentalis* Hook.f. | 53.63 | 20.30 | 3.90 | 9.50 | 4.37 | 92.50 | Ajayi 2009 |
| *Plukenetia conophora* Müll.Arg. | 4.28 | 21.65 | 5.27 | 7.34 | 19.96 | 58.5 | Ayoola et al. 2011 |
| *Treculia africana* Decne. ex Trécul | 14.68 | 17.10 | 3.01 | 2.91 | 57.00 | 90.53 | Arigbede et al. 2008 |
| *Tetracarpidium conophorum* (Müll.Arg.) Hutch. & Dalziel | 3.00 | 52.60 | 6.00 | 12.70 | 6.50 | 97.60 | Clergé Tchiegang et al. 2006 |
| *Vitellaria paradoxa* C.F.Gaertn. | 30.49 | 8.46 | 4.35 | 9.99 | 45.92 | 96.90 | Honfo et al. 2014; Raimi et al. 2014; Ugese et al. 2010 |
| *Xylopia aethiopica* (Dunal) A.Rich. | 17.96 | 7.30 | 7.52 | 20.96 | 28.79 | 88.60 | Abolaji et al. 2007; Bouba et al. 2012; Dike 2010 |
| *Zanthoxylum zanthoxyloides* (Lam.) Zepern. & Timler | 19.30 | 8.00 | 9.50 | 5.00 | 6.00 | 90.40 | Bouba et al. 2012 |
