# Supplementary material for: Carbohydrates, proteins, fats and other essential components of food from native trees in West Africa
Source: Heliyon. 2019 May 22;5(5):e01744. doi: 10.1016/j.heliyon.2019.e01744 (PMC6531672; doi:10.1016/j.heliyon.2019.e01744)
Supplement: Appendix 3 [file mmc3.docx]

Appendix 3. Average values of proximate composition in fruit parts (apart from the seed)

| Species | Fat | Protein | Ash | Fiber | Carbohydrate | Dry matter | Authors |
| --- | --- | --- | --- | --- | --- | --- | --- |
| *Adansonia digitata* L. | 0.74 | 3.27 | 5.50 | 12.54 | 78.25 | 91.09 | Assogbadjo et al. 2012; Greene 1932; Lockett et al. 2000; Makalao et al. 2015; Magaia et al. 2013; Murray et al. 2001; Osman 2004; Stadlmayr et al. 2013; Edwige et al. 2014 |
| *Anisophyllea laurina* R.Br. ex Sabine | 1.31 | 1.56 | 5.72 | 2.43 | 88.98 | 17.99 | Onivogui et al. 2014 |
| *Annona senegalensis* Pers. | 1.10 | 1.05 | 1.10 | 9.75 | 50.00 | 23.00 | Herzog et al. 1994 |
| *Balanites aegyptiaca* (L.) Delile | 0.97 | 6.04 | 5.71 | 5.565 | 70.95 | 86.80 | Lockett et al. 2000; Stadlmayr et al. 2013 |
| *Borassus aethiopum* Mart. | 1.74 | 4.25 | 4.26 | 18.60 | 64.07 | 92.92 | Lockett et al. 2000 |
| *Bridelia ferruginea* Benth. | 8.91 | 7.31 | 3.12 | 28.12 | 44.81 | 92.28 | Lockett et al. 2000 |
| *Canarium schweinfurthii* Engl. | 29.62 | 1.84 | 6.12 | 1.88 | 60.54 | 91.05 | Dike 2010 |
| *Carpolobia lutea* G.Don | 1.99 | 9.19 | 3.74 | 1.04 | 84.04 | 90.38 | Dike 2010 |
| *Chrysophyllum albidum* G. Don | 6.97 | 6.03 | 3.79 | 8.70 | 71.96 | 61.39 | Edem et al. 1984; Dike 2010 |
| *Cola pachycarpa* K.Schum. | 2.73 | 9.10 | 12.84 | 7.55 | 67.79 | 34.95 | Essein et al. 2017 |
| *Cordia sinensis* Lam. | 1.80 | 12.60 | 5.20 | 11.60 | 50.00 | 27.00 | Murray et al. 2001 |
| *Dacryodes edulis* (G.Don) H.J.Lam | 20.47 | 7.85 | 2.64 | 1.36 | 46.68 | 49.37 | Dike 2010; Okwu and Nnamdi 2008; Stadlmayr et al. 2013 |
| *Dennettia tripetala* Baker f. | 6.57 | 10.73 | 4.96 | 10.13 | 65.06 | 91.45 | Dike 2010; Okwu and Morah 2004 |
| *Detarium microcarpum* Guill. & Perr. | 1.60 | 19.22 | 2.84 | 20.40 | 51.55 | 94.21 | Lockett et al. 2000; Mariod et al. 2009; Edwige et al. 2014 |
| *Dialium guineense* Willd. | 3.75 | 6.25 | 2.50 | 1.40 | 82.80 | 95.55 | Achoba et al. 1992; Oladejo 2009 |
| *Ficus sycomorus* L. | 7.09 | 7.50 | 9.18 | 34.77 | 41.47 | 12.53 | Lockett et al. 2000 |
| *Afraegle paniculata* (Schumach. & Thonn.) Engl. | 0.33 | 4.19 | 3.59 | 9.12 | 76.86 | 94.09 | Lockett et al. 2000 |
| *Garcinia kola* Heckel | 3.25 | 1.28 | 3.43 | 2.98 | 46.38 | 52.90 | Chinonyerem et al. 2017; Dike 2010 |
| *Gardenia erubescens* Stapf & Hutch. | 1.37 | 3.39 | 2.74 | 22.41 | 38.70 | 52.28 | Bello et al. 2008; Edwige et al. 2014 |
| *Grewia betulaefolia* Baill. | 2.00 | 12.00 | 6.70 | 13.20 | 50.00 | 74.00 | Murray et al. 2001 |
| *Irvingia gabonensis* (Aubry-Lecomte ex O'Rorke) Baill. | 1.10 | 1.10 | 0.80 | 0.40 | 17.80 | 21.20 | Stadlmayr et al. 2013 |
| *Landolphia hirsuta* (Hua) Pichon | 3.37 | 2.45 | 2.67 | 6.73 | 42.38 | 41.39 | Dike 2010; Herzog et al. 1994 |
| *Landolphia owariensis* P.Beauv. | 0.50 | 0.85 | 0.50 | 5.80 | 37.50 | 20.95 | Herzog et al. 1994 |
| *Lannea schimperi* (Hochst. ex A.Rich.) Engl. | 0.76 | 7.76 | 4.23 | 43.44 | 43.80 | 5.58 | Lockett et al. 2000 |
| *Mondia whitei* (Hook.f.) Skeels | 1.60 | 6.20 | 6.80 | 11.80 | 2.10 | 28.50 | Oladejo 2009 |
| *Parinari curatellifolia* Planch. ex Benth. | 1.53 | 7.22 | 4.22 | 58.77 | 28.27 | 7.28 | Lockett et al. 2000 |
| *Parkia biglobosa* (Jacq.) G.Don | 5.36 | 5.46 | 6.24 | 16.42 | 58.51 | 69.31 | Alabi et al. 2005; Bello et al. 2008; Gernah et al. 2007; Lockett et al. 2000; Edwige et al. 2014 |
| *Saba comorensis* (Bojer ex A.DC.) Pichon | 10.50 | 0.13 | 7.30 | 9.10 | 72.40 | 98.50 | Omale et al. 2010 |
| *Saba senegalensis* (A.DC.) Pichon | 8.92 | 0.53 | 2.80 | 13.52 | 74.23 | 98.50 | Boamponsem et al. 2013 |
| *Sarcocephalus latifolius* (Sm.) E.A.Bruce | 0.40 | 1.50 | 1.20 | 17.90 | 4.00 | 28.50 | Herzog et al. 1994 |
| *Sclerocarya birrea* (A.Rich.) Hochst. | 0.50 | 3.60 | 6.80 | 37.70 | 17.32 | 17.00 | Murray et al. 2001 |
| *Synsepalum dulcificum* (Schumach. & Thonn.) Daniell | 2.10 | 2.48 | 0.87 | 0.57 | 48.85 | 54.88 | Njoku et al. 2016 |
| *Syzygium guineense* (Willd.) DC. | 0.70 | 1.60 | 1.00 | 1.80 | 13.50 | 18.50 | Stadlmayr et al. 2013 |
| *Tamarindus indica* L. | 1.09 | 5.57 | 2.58 | 6.97 | 64.01 | 65.04 | El-Siddig et al. 2006; Ishola et al. 1990; Nordeide et al. 1996; Saka and Msonthi 1994; Stadlmayr et al. 2013 |
| *Tetrapleura tetraptera* (Schum. & Thonn.) Taub. | 7.69 | 8.08 | 8.09 | 17.29 | 37.64 | 89.23 | Bouba et al. 2012; Dike 2010; Ene-Obong et al. 2016 |
| *Treculia africana* Decne. ex Trécul | 9.08 | 17.57 | 2.64 | 2.91 | 57.00 | 89.19 | Appiah 2011 |
| *Vitellaria paradoxa* C.F.Gaertn. | 1.78 | 4.13 | 5.55 | 25.78 | 29.70 | 55.28 | Aguzue et al. 2013; Honfoet al. 2014; Okullo et al. 2010; Ugese et al. 2008 |
| *Vitex doniana* Sweet | 1.24 | 1.63 | 1.09 | 1.40 | 26.30 | 22.47 | Makalao et al. 2015; Stadlmayr et al. 2013 |
| *Ximenia americana* L. | 28.23 | 15.21 | 4.13 | 27.54 | 24.90 | 4.25 | Lockett et al. 2000 |
| *Ziziphus mauritiana* Lam*.* | 1.09 | 4.84 | 3.62 | 7.50 | 64.21 | 33.56 | Lockett et al. 2000; Chandra et al. 1994; Makalao et al. 2015; Nyanga et al. 2013; Stadlmayr et al. 2013 |
