# Supplementary material for: Carbohydrates, proteins, fats and other essential components of food from native trees in West Africa
Source: Heliyon. 2019 May 22;5(5):e01744. doi: 10.1016/j.heliyon.2019.e01744 (PMC6531672; doi:10.1016/j.heliyon.2019.e01744)
Supplement: Appendix 4 [file mmc4.docx]

Appendix 4. Average values of proximate composition in leaves

| Species | Fat | Protein | Ash | Fiber | Carbohydrate | Dry matter | Authors |
| --- | --- | --- | --- | --- | --- | --- | --- |
| *Adansonia digitata* L. | 8.02 | 13.06 | 11.55 | 14.96 | 65.41 | 64.91 | Assogbadjo et al. 2012; Dike 2010; Lockett et al. 2000; Edwige et al. 2014 |
| *Afzelia africana* Pers. | 5.29 | 11.43 | 7.33 | 31.11 | 44.84 | 91.35 | Dike 2010 |
| *Albizia glaberrima* (Schum. & Thonn.) Benth. | 2.28 | 25.31 | 7.58 | 21.46 | 24.55 | 80.99 | Olujobi 2015 |
| *Baphia pubescens* Hook.f. | 6.59 | 12.00 | 11.85 | 12.36 | 45.27 | 50.00 | Ogunwa et al. 2016 |
| *Blighia unijugata* Baker | 3.80 | 24.13 | 6.02 | 12.61 | 41.31 | 87.87 | Offor et al. 2014 |
| *Bombax buonopozense* P.Beauv. | 2.70 | 14.17 | 7.74 | 15.57 | 39.86 | 79.64 | Olujobi 2015 |
| *Ceiba pentandra* (L.) Gaertn. | 9.40 | 18.80 | 10.40 | 21.69 | 34.41 | 94.70 | Raimi et al. 2014 |
| *Cissus populnea* Guill. & Perr. | 9.60 | 13.30 | 11.40 | 9.00 | 57.24 | 30.90 | Adebowale et al. 2015 |
| *Ficus glumosa* Delile | 5.23 | 51.67 | 6.23 | 1.79 | 14.4 | 72.2 | Agiang et al. 2016 |
| *Ficus thonningii* Blume | 7.55 | 13.90 | 9.43 | 28.87 | 30.92 | 33.04 | Otitoju et al. 2014; Lockett et al. 2000 |
| *Gongronema latifolium* Benth. | 9.42 | 23.40 | 6.23 | 8.61 | 52.30 | 86.89 | Balogun et al. 2016; Dike 2010 |
| *Grewia carpinifolia* Juss. | 5.19 | 18.70 | 9.92 | 16.10 | 50.00 | 93.68 | Adbiyi et al. 2015 |
| *Hymenocardia ulmoides* Oliv. | 2.75 | 15.12 | 3.29 | 2.00 | 8.57 | 32.80 | Andzouana and Mombouli. 2011 |
| *Lecaniodiscus cupanioides* Planch. ex Benth. | 5.22 | 14.85 | 6.94 | 9.02 | 55.55 | 89.06 | Dike 2010; Olujobi 2015 |
| *Maerua angolensis* DC. | 3.12 | 33.21 | 12.90 | 14.98 | 28.43 | 91.77 | Emmanuel et al. 2011 |
| *Myrianthus arboreus* P.Beauv. | 1.32 | 12.49 | 6.61 | 10.00 | 14.31 | 42.08 | Otitoju et al. 2014 |
| *Opilia amentacea* Roxb. | 2.45 | 14.8 | 21.09 | 16.06 | 34.94 | 91.40 | Emmanuel et al. 2011 |
| *Pterocarpus mildbraedii* Harms | 6.46 | 22.53 | 13.19 | 16.81 | 34.345 | 89.02 | Akinyeye et al. 2010; Dike 2010 |
| *Pterocarpus santalinoides* DC. | 4.08 | 24.62 | 7.42 | 19.99 | 26.09 | 66.67 | Agiang et al. 2016; Fasae et al. 2010; Ndukwe 2013; Otitoju et al. 2014 |
| *Pterocarpus soyauxii* Taub. | 4.73 | 17.57 | 8.30 | 15.25 | 48.48 | 89.85 | Dike 2010; Ndukwe 2013 |
| *Sterculia tragacantha* Lindl. | 2.62 | 18.04 | 11.80 | 22.16 | 33.72 | 88.02 | Olujobi 2015 |
| *Tamarindus indica* L. | 3.90 | 14.00 | 5.50 | 14.00 | 72.70 | 96.10 | Nordeide et al. 1996 |
| *Telfairia occidentalis* Hook.f. | 5.58 | 20.16 | 8.54 | 8.85 | 57.06 | 90.44 | Dike 2010; Fasuyi and Nonyerem 2007; Usunobun and Egharebva 2014 |
| *Vernonia amygdalina* Delile | 8.07 | 25.29 | 7.37 | 13.13 | 65.90 | 91.64 | Belewu et al. 2009; Dike 2010 |
| *Vitex doniana* Sweet | 2.67 | 25.63 | 8.64 | 18.23 | 28.15 | 76.63 | Agiang et al. 2016; Olujobi 2015; Otitoju et al. 2014 |
