# Supplementary material for: Carbohydrates, proteins, fats and other essential components of food from native trees in West Africa
Source: Heliyon. 2019 May 22;5(5):e01744. doi: 10.1016/j.heliyon.2019.e01744 (PMC6531672; doi:10.1016/j.heliyon.2019.e01744)
Supplement: Appendix 5 [file mmc5.docx]

Appendix 5. Species abbreviations

Acacmacr: *Acacia macrostachya*, Adandigi: *Adansonia digitata*, Afrapani: *Afraegle paniculata*, Afrolepi: *Afrostyrax lepidophyllus*, Afzeafri: *Afzelia Africana*, Afzebell: *Afzelia bella*, Albiglab: *Albizia glaberrima*, Annosene: *Annona senegalensis*, Balaaegy: *Balanites aegyptiaca*, Baphpube: *Baphia pubescens*, Bligsapi: *Blighia sapida*, Bligunij: *Blighia unijugata*, Bombbuon: *Bombax buonopozense*, Boraaeth: *Borassus aethiopum*, Boscsene: *Boscia senegalensis*, Braceury: *Brachystegia eurycoma*, Bracnige: *Brachystegia nigerica*, Bridferr: *Bridelia ferruginea*, Buchcori: *Buchholzia coriacea*, Canaschw: *Canarium schweinfurtii*, Carplute: *Carpolobia lutea*, Ceibpent: *Ceiba pentandra*, Chryalbi: *Chrysophyllum albidum*, Cisspopu: *Cissus populnea*, Colaacum: *Cola acuminata*, Colamill: *Cola millenii*, Colaniti: *Cola nitida*, Colapach: *Cola pachycarpa*, Cordsine: *Cordia sinensis*, Dacredul: *Dacryodes edulis*, Daniogea:*Daniellia ogea*, Danioliv: *Daniellia oliveri*, Denntrip: *Dennettia tripetala*, Detamicr: *Detarium microcarpum*, Dialguin: *Dialium guineense*, Dichcine: *Dichrostachys cinerea*, Diosmesp: *Diospyros mespiliformis*, Ficuglum: *Ficus glumosa*, Ficusyco: *Ficus sycomorus*, Ficuthon: *Ficus thonningii*, Garckola: *Garcinia kola*, Garderub: *Gardenia erubescens*, Gonglati: *Gongronema latifolium*, Grewbetu: *Grewia betulaefolia*, Grewcarp: *Grewia carpinifolia*, Hymeulmo: *Hymenocardia* *ulmoides*, Irvigabo: *Irvingia gabonensis*, Landowar: *Landolphia owariensis*, Landtogo: *Landolphia togolana*, Lannmicr: *Lannea microcarpa*, Lannschi: *Lannea schimperi*, Lecacupa: *Lecaniodiscus cupanioides*, Lophlanc: *Lophira lanceolata*, Maerango: *Maerua angolensis*, Mondwhit: *Mondia whitei*, Monomyri: *Monodora myristica*, Mucusloa: *Mucuna sloanei*, Myriarbo: *Myrianthus arboreus*, Olaxsubs: *Olax subscorpioides*, Opilamen: *Opilia amentacea*, Pachglab: *Pachira glabra*, Paricura: *Parinari curatellifolia*, Pariexce: *Parinari excelsa*, Parkbigl: *Parkia biglobosa*, Pentmacr: *Pentaclethra macrophylla*, Plukcono: *Plukenetia conophora*, Prosafri: *Prosopis africana*, Ptermild: *Pterocarpus mildbraedii*, Ptersant: *Pterocarpus santalinoides*, Ptersoya: *Pterocarpus soyauxii*, Riciheud: *Ricinodendron heudelotii*, Sabacomo: *Saba comorensis*, Sabasene: *Saba senegalensis*, Salvpers: *Salvadora persica*, Sarclati: *Sarcocephalus latifolius*, Sclebirr: *Sclerocarya birrea*, Scorzenk: *Scorodophloeus zenkeri*, Sphesten: *Sphenostylis stenocarpa*, Sterafri: *Sterculia africana*, Stertrag: *Sterculia tragacantha*, Synsdulc: *Synsepalum dulcificum*, Syzyguin: *Syzygium guineense*, Tamaindi: *Tamarindus indica*, Telfocci: *Telfairia occidentalis*, Termseri: *Terminalia sericea*, Tetrtetr: *Tetrapleura tetraptera*, Trecafri: *Treculia africana*, Vernamyg: *Vernonia amygdalina*, Vitedoni: *Vitex doniana*, Vitemomb: *Vitex mombassae*, Vitepara: *Vitellaria paradoxa*, Ximeamer: *Ximenia americana*, Xyloaeth: *Xylopia aethiopica*, Zantzant: *Zanthoxylum zanthoxyloides*, Zizimaur: *Ziziphus mauritiana*

**Appendices References**

Abolaji, A. O., Adebayo, H. A., & Odesanmi, O. S. (2007). Nutritional qualities of three medicinal plant parts (*Xylopia aethiopica*, *Blighia sapida* and *Parinari polyandra*) commonly used by pregnant women in the western part of Nigeria. *Pakistan Journal of Nutrition,* *6***,** 665-668.

Achi O. K., & Okolo N. I. (2004). The chemical composition and some physical properties of a water-soluble gum from *Prosopis africana* seeds. *International Journal of Food Science and Technology,* *39***,** 431-436. doi:10.1111/j.1365-2621.2004.00801.x

Achoba, I. I., Lori, J. A., Elegbede, J. A., & Kagbu, J. A. (1992). Nutrient composition of black (African) velvet tamarind (*Dialium guineense* Wild) seed and pulp from Nigeria. *Journal of Food Biochemistry*, *16*, 229-233.

Adebiyi, O. E., Soetan, K.O., & Olayemi, O. F. (2015). Comparative studies on the proximate compositions, minerals and anti-nutritional factors in the leaves and stem of *Grewia carpinifolia*. *Annals,* *Food Science and Technology*, *16*, 207-217.

Adebowale, K. O., Nwokocha, L. M., & Agbaje, W. B. (2015). Composition and food value of leaves of two tropical food thickeners – *Bombax costatum* and *Cissus populnea*. *Canadian Journal of Pure and Applied Sciences,* *9*, 3221-3227.

Agiang, M., Mgbang, J., Essien, N., & Peters, H. (2016). Proximate and phytochemical composition of some lesser known leafy vegetables consumed in northern Senatorial District of Cross River State, Nigeria. *World Journal of Nutrition and Health,* *4***,** 16-21. DOI:10.12691/jnh-4-1-4

Aguzue, O. C., Akanji, F. T., Tafida, M. A., & Kamal, M. J. (2013). Nutritional and some elemental composition of Shea (V*itellaria paradoxa*) fruit pulp. *Archives of Applied Science Research,* *5***,** 63-65.

Ajah, P. O., & Madubuike, F. N. (1997). The proximate composition of some tropical legume seeds grown in two states in Nigeria. *Food Chemistry,* *59***,** 361-365.

Ajayi, A. I. (2009). Proximate and mineral element composition of seven selected unexploited oilseeds from Nigeria. *Food,* *3***,** 65-67.

Ajayi, I. A., Oderinde, R. A., Kajogbola, D. O., & Uponi, J. I. (2006). Oil content and fatty acid composition of some underutilized legumes from Nigeria. *Food Chemistry,* *99***,** 115-120.

Akanni, M. S., Adekunle, A. S., & Oluyemi, E. A. (2005). Physicochemical properties of some non-conventional oilseeds. *Journal of Food Technology,* *3***,** 177-181.

Akinyeye, R.O., Oluwadunsin, A., & Omoyeni, A. (2010). Proximate, mineral, anti-nutrients, phyto-chemical screening and amino acid compositions of the leaves of *Pterocarpus mildbraedi* Harms. *Electronic Journal of Environmental, Agricultural and Food Chemistry,* *9***,** 1322-1333.

Akoja, S. S., & Amoo, I. A. (2011). Proximate composition of some under-exploited leguminous crop species. *Pakistan Journal of Nutrition*, *10***,** 143-146.

Akpata, M. I., & Miachi, O. E. (2001). Proximate composition and selected functional properties of Detarium microcarpum. *Plant Foods for Human Nutrition,* *56***,** 297-302.

Alabi, D. A., Akinsulire, O. R., & Sanyaolu, M. A. (2005). Qualitative determination of chemical and nutritional composition of Parkia biglobosa (Jacq.) Benth. *African Journal of Biotechnology,* 4, 812-815.

Amoo, I. A., & Atasie, V. N. (2012). Nutritional and functional properties of Tamarindus indica pulp and Zizyphus spina-christi fruit and seed. *Journal of Food Agriculture and Environment,* 10**,** 16-19.

Appiah, F. 2011. Nutrient composition, functional properties, digestibility and formulation of selected food products from breadfruits (*Artocarpus* spp. and *Treculia africana*). PhD Dissertation in Food Science and Technology, Kwame Nkrumah University of Science and Technology Kumasi, Ghana.

Arigbede, O. M., Anele, U. Y., Jolaosho, A. O., Olanite, J. A., Onifade, O. S., & Wahab, T. A. (2008). Chemical composition and in vitro gas production of african bread fruit (*Treculia africana*) var. Decne. *Archivos de Zootecnia,* *57***,** 113-121.

Assogbadjo, A. E., Chadare, F. J., kakaï, R. G., Fandohan, B., & Baidu-Forson, J. J. (2012). Variation in biochemical composition of baobab (*Adansonia digita*) pulp, leaves and seeds in relation to soil types and tree provenances. *Agriculture, Ecosystems and Environment,* *157***,** 94-99. https://doi.org/10.1016/j.agee.2012.01.021

Ayoola, P. B., Anawumi, O. O., & Faboya, O. O. P. (2011). Chemical evaluation and nutritive values of Tetracarpidium conophorum (Nigerian walnut) seeds. *Journal of Pharmaceutical and Biomedical Sciences,* *11***,** 1-5.

Balogun, B. I. (2013). Evaluation of the nutritional potentials of fermented oil beans seed *Pentaclethra macrophyllah* Benth. *Production Agriculture and Technology,* *9***,** 73-87.

Balogun, M. E., Besong, E. E., Obimma, J. N., Mbamalu, O. S., & Djobissie, S. F. A. (2016). *Gongronema latifolium*: A phytochemical, nutritional and pharmacological review. *Journal of Physiology and Pharmacology Advances,* *6*, 811-824. OI: 10.5455/jppa.1969123104000

Bamidele, O. P., Ojedokoun, S. O., & Fasogbon, B. M. (2015). Physico-chemical properties of instant ogbono (*Irvingia gabonensis*) mix powder. *Food Science and Nutrition,* *3***,** 313-318. doi: 10.1002/fsn3.220

Barminas, J. T., Maina, H. M., & Ali, J. (1998). Nutrient content of *Prosopis africana* seeds. *Plant Foods for Human Nutrition,* 52, 325-328. https://doi.org/10.1023/A:1008045218320

Batista, G. A., Esteves, A. E., Dessimoni-Pinto, A. V. N., Oliveira, G. L., Pires, T. S., & Santana, C. R. (2011). Chemical composition of Jatobá-do-cerrado (*Hymenaea stigonocarpa* Mart.) flour and its effect on growth of rats. *Alimentos E Nutrição, Araraquara,* *22*, 173-180.

Belewu, M. A., Olatunde, O. A., & Giwa, T. A. (2009). Underutilized medicinal plants and spices: Chemical composition and phytochemical properties. *Journal of Medicinal Plants Research,* 3, 1099-1103.

Bello, M. O., Falade, O. S., Adewusi, S. R. A., & Olawore, N. O. (2008). Studies on the chemical compositions and anti-nutrients of some lesser known Nigeria fruits. *African Journal of Biotechnology*, *7*, 3972-3979.

Bertin, R., Labronici, G. L., Valdemiro, d. S. G.C. B., Azevedo, M. S., Maltez, H. F., Melina, H., Gustavo, M. A., Tavares L. B. B., & Roseane, F. (2014). Nutrient composition and, identification/quantification of major phenolic compounds in *Sarcocornia ambigua* (Amaranthaceae) using HPLC–ESI-MS/MS. *Food Research International*, *55*, 404-411. https://doi.org/10.1016/j.foodres.2013.11.036

Boamponsem, G. A., Johnson, F. S., Mahunu, G. K., & Awiniboya S. F. (2013). Determination of biochemical composition of *Saba senegalensis* (Saba fruit). *Asian Journal of Plant Science and Research,* **3**, 31-36.

Bouba, A. A., Njintang, N. Y., Foyet, H. S., Scher, J., Montet, D., & Mbofung, C. M. F. (2012). Proximate composition, mineral and vitamin content of some wild plants used as spices in Cameroon. *Food and Nutrition Sciences,* *3***,** 423-432.

Chinedu, S. N., & Nwinyi, C. O. (2012). Proximate analysis of *Sphenostylis stenocarpa* and *Voadzeia subterranean* consumed in South –Eastern Nigeria. *Journal of Agricultural Extension and Rural Development* *4*, 57-62. https://doi.org/10.5897/JAERD11.031

Chinonyerem, A. N., Obioha, O., & Blessing, A. U. (2017). *Garcinia kola* fruit pulp: Evaluation of it's nutrient, phytochemical and physicochemical properties. *Journal of Applied Life Sciences International*, *13*, 1-10. DOI: 10.9734/JALSI/2017/33558

Chivandi, E. (2012). In vitro and in vivo chamical characterization of *Kigelia Africana*, *Mimusops zeyheri*, *Terminalia sericea* and *Ximenia caffra* nut and nut meals. PhD Dissertation at University of the Witwatersrand, Johannesburg.

Chouaibi, M., Mahfoudhi, N., Rezig, L., Donsì, F., Ferrari, G., & Hamdi, S. (2012). Nutritional composition of *Zizyphus lotus* L. seeds. *Journal of the Science and Agriculture*, *92*, 1171-1177.

Clergé Tchiegang, L. B., Kenfack, M., Tenin, D., & Ndjouenkeu, R. (2006). Physicochemical and functional properties of defatted cakes from two Euphorbiaceae from Cameroon: *Riconodendron heudelotii* (Bail) and *Tetracarpidium conophorum* (Müll. Arg.). *Journal of Food Technology,* *4*, 96-100.

Dah-Nouvlessounon, D., Adjanohoun, A., Sina, H., Noumavo, P. A., Diarrasouba, N., Parkouda, C., Madodé, Y. E., Dicko, M. H., & Baba-Moussa, L. (2015). Nutritional and anti-nutrient composition of three kola nuts (*Cola nitida*, *Cola acuminata* and *Garcinia kola*) Produced in Benin. *Food and Nutrition Sciences,* *6*, 1395-1407. http://dx.doi.org/10.4236/fns.2015.615145

Dike, M. C. (2010). Proximate, phytochemical and nutrient compositions of some fruits, seeds and leaves of some plant species at Umudike, Nigeria. *Journal of Agricultural and Biological Science,* *5*, 7-16.

Dosumu, O. O., Oluwaniyi, O. O., Awolola, G. V., & Oyedeji, O. O. (2012). Nutritional composition and antimicrobial properties of three Nigerian condiments. *Nigerian Food Journal*, *30*, 43-52.

Ebana, R. U. B., Edet, U. O., Ekanemesang, U. M., Ikon, G. M., Umoren, E. B., Ntukidem, N. W., Etim, O. E., Sambo, S., & Brown, N. U. (2017). Proximate composition and nutritional analysis of seeds and testas of *Dacryodes edulis* and *Garcinia kola*. *Asian Journal of Biology,* *2*, 1-8. DOI: 10.9734/AJOB/2017/31159

Edem, D. O., Eka, O. U., & Ifon, E. T. (1984). Chemical evaluation of nutritive value of the fruit of African starapple (*Chrysophyllum albidum*). *Food chemistry,* *14*, 303-311.

Edwige, T. D. N., Charles, P., Niéyidouba, L., Aminata, S., Margarida, C. E. A., & Joseph, B. I. (2014). Nutritional composition of five food trees species products used in human diet during food shortage period in Burkina Faso. *African Journal of Biotechnology,* *13*, 1807-1812. http://dx.doi.org/10.5897/AJB2013.13462

Effiom, E. O., Okon, B. I., & Okon, I. R. (2016). Proximate and mineral analysis of methanolic leaf extract of *Napoleona imperialis* (mkpodu). *International Journal of Biochemistry Research & Review,* 10, 1-8. OI: 10.9734/IJBCRR/2016/23352

Elbadawi, S. M. A., Ahmad, E. E. M., Mariod, A. A., & Mathäus, B. (2017). Effects of thermal processing on physicochemical properties and oxidative stability of *Balanities aegyptiaca* kernels and extracted oil. *Grasas y Aceites*, *68*, 184. doi: http://dx.doi.org/10.3989/gya.1048162

El-Siddig, K., Gunasena, H. P. M., Prasa, B. A., Pushpakumara, D. K. N. G., Ramana, K. V. R., Vijayanand, P., & Williams, J. T. (2006). Tamarind – *Tamarindus indica* L. Fruits for the Future 1. Southampton Centre for Underutilized Crops, Southampton, UK.

Emmanuel, T. V., Njoka, J. T., Catherine, L. W., & Lyaruu, H. V. (2011). Nutritive and anti-nutritive qualities of mostly preferred edible woody plants in selected drylands of Iringa District, Tanzania. *Pakistan Journal of Nutrition,* *10*, 786-791.

Ene-Obong, H., Onuoha, N., Aburime, L., & Mbah, O. (2018). Chemical composition and antioxidant activities of some indigenous spices consumed in Nigeria. *Food Chemistry,* *238*, 58-64. https://doi.org/10.1016/j.foodchem.2016.12.072

Eromosele, I. C., & Eromosele, C. O. (1993). Studies on the chemical composition and physico-chemical properties of seeds of some wild plants. *Plant Foods for Human Nutrition (Formerly Qualitas Plantarum),* *43*, 251-258.

Eromosele, I. C., Eromosele, C. O., Akintoye, A. O., & Komolafe, T. O. (1994). Characterization of oils and chemical analyses of the seeds of wild plants. *Plant Foods for Human Nutrition (Formerly Qualitas Plantarum),* *46*, 361-365.

Essien, E. E., & Udousoro, I. I. (2017). *Cola parchycarpa* K. Schum: Chemical evaluation of amino acids, vitamins and other nutritional factors in seed, fruit mesocarp and epicarp. *UK Journal of Pharmaceutical and Biosciences,* *5*, 23-29.

Ezeagu, I. E. (2017). Baobab (*Adansonia digitata* L.) seed protein utilization in young albino rats I: biochemical ingredients and performance characteristics. *Animal Research International*, *2*(1).

Ezeagu, I. E., Metges, C. C., Proll, J., Petzke, K. J., & Akinsoyinu, A. O. (1996). Chemical composition and nutritive value of some wild-gathered tropical plant seeds. *Food and Nutrition Bulletin-United Nations University,* *17*, 275-278.

Fasae, O. A., Sowande, O. S., & Popoola, A. A. (2010). Evaluation of selected leaves of trees and foliage of shrubs as fodder in ruminant production. *Journal of Agricultural Science and Environment,* *10*, 36-44.

Fasuyi, A. O., & Nonyerem, A. D. (2007). Biochemical, nutritional and haematological implications of *Telfairia occidentalis* leaf meal as protein supplement in broiler starter diets. *African Journal of Biotechnology,* *6*, 1055-1063.

Gbadamosi, I. T., Moody, J. O., & Yekini, A. O. (2012). Nutritional composition of ten ethnobotanicals used for the treatment of anaemia in Southwest Nigeria. *European Journal of Medicinal Plants,* *2*, 140-150

Gernah, D. I., Atolagbe, M. O., & Echegwo, C. C. (2007). Nutritional composition of the African locust bean (*Parkia biglobosa*) fruit pulp. *Nigerian Food Journal,* *25*, 190-196.

Greene, R. A. (1932). Composition of the pulp and seeds of *Adansonia digitata*. *Botanical Gazette,* *94*, 215-220.

Herzog, F., Farrah, Z., & Amado, R. (1994). Composition and consumption of gathered wild fruits in the V-Baoulé Côte d’Ivoire. *Ecology of Food and Nutrition,* *32*, 181-196.

Igwenyi, I. O., & Azoro, B. N. (2014). Proximate and phytochemical compositions of four indigenous seeds used as soup thickeners in ebonyi state Nigeria. *Journal of Environmental Science, Toxicology and Food Technology,* *8*, 35-40.

Ijarotimi, O. S., Fagbemi, T.N., & Faramade, O. O. (2015). Determination of chemical composition, nutritional quality and anti-diabetic potential of raw, blanched and fermented wonderful kola (*Bucholzia coriacea*) Seed Flour. *Journal of Human Nutrition & Food Science,* *3*, 1060.

Ishola, M. M., Agbaji, E. B., & Agbaji, A. S. (1990). A chemical study of *Tamarindus indica* (tsamiya) fruits grown in Nigeria. *Journal of the Science of Food and Agriculture,* 51, 141-143.

Jimoh, F. O., & Oladiji, A. T. (2005). Preliminary studies on *Piliostigma thonningii* seeds: Proximate analysis, mineral composition and phytochemical screening. *African Journal of Biotechnology,* *4*, 1439-1442.

Kyari, B. A., Mohammed, F. K., Apagu, B., & Waziri, M. S. (2017). The relationship between seed weight, nutrient composition and germinating seeds of five tree species of the Lake Chad Basin area of Nigeria. *Donnish Journal of Agricultural Research,* *4*, 001-004.

Lockett, C. T., Calvert, C. C., & Grivetti, L. E. (2000). Energy and micronutrient composition of dietary and medicinal wild plants consumed during drought. Study of rural Fulani, Northeastern Nigeria. *International Journal of Food Sciences and Nutrition,* *51*, 195-208.

Lohlum, S. A., Maikidi, G. H., & Solomon, M. (2010). Proximate composition, amino acid profile and phytochemical screening of *Lophira lanceolata* seeds. *African Journal of Food, Agriculture, Nutrition and Development,* *10*, 2012-2023.

Magaia, T., Uamusse, A., Sjöholm, I., & Skog, K. (2013). Proximate analysis of five wild fruits of Mozambique. *The Scientific World Journal,* 1-7. http://dx.doi.org/10.1155/2013/601435

Makalao, M. M., Savadogo, A., Zongo, C., & Traore, A. S. (2015). Composition nutritionnelle de 10 fruits sauvages consommés dans trois départements du Tchad. *International Journal of Biological and Chemical Sciences,* *9*, 2385-2400. DOI : http://dx.doi.org/10.4314/ijbcs.v9i5.11

Mariod, A., Mirghani, M., Abdul, A., & Abdelwahab, S. (2009). *Detarium microcarpum* Guill and Perr fruit proximate chemical analysis and sensory characteristics of concentrated juice and jam. *African Journal of Biotechnology, 8*(17).

Muhammad, S., Hassan, L. G., Dangoggo, S. M., Hassan, S. W., Umar, K. J., & Aliyu, R. U. (2011). Nutritional and antinutritional composition of *Sclerocarya birrea* seed kernel. *Studia Universitatis Vasile Goldis Seria Stiintele Vietii (Life Sciences Series),* *21*(4).

Murray, S. S., Schoeninger, J. M., Bunn, T. H., Pickering, R. T., & Marlett, A. J. (2001). Nutritional composition of some wild plant foods and honey used by hadza foragers of Tanzania. *Journal of Food Composition and Analysis,* *14***,** 3-13. doi:10.1006/jfca.2000.0960

Musa, H., & Bichi, A.H. (2015). Effect of different heat processing methods on the proximate composition of *Piliostegma reticulatum* seed meal. *Bayero Journal of Pure and Applied Sciences.* *8*, 115-122. http://dx.doi.org/10.4314/bajopas.v8i1.20

Nduche, M. U., Edeoga, H. O., Omosun, G., & Nwankwo, D. (2015). Evaluation of the chemical composition of five Nigerian medicinal plants. *IOSR Journal of Pharmacy and Biological Sciences (IOSR-JPBS).* *10*, 27-31. DOI: 10.9790/3008-10232731

Ndukwe, O. K., & Ikpeama, A. (2013). Comparative evaluation of the phytochemical and proximate constituents of OHA (*Pterocarpus Soyansii*) and Nturukpa (*Pterocarpus Santalinoides*) leaves. *International Journal of Academic Research in Progressive Education and Development,* *2*, 22-31. http://dx.doi.org/10.6007/IJARPED/2-i3/22

Njoku, N. E., Ubbaonu, C. N., Alagbaoso, S. O., Agunwa, I. M, & Eluchie, C. N. (2016). Proximate, anti-nutritional and phytochemical composition of the yellow variety of the *Synsepalum dulcificum* (miracle fruit) Berry. *American Journal of Food Science and Technology,* *4*, 102-108. DOI:10.12691/ajfst-4-4-3

Njoku, O. U., Obioma, U., & Frank, E. U. (1999). Investigation on some nutritional and toxicological properties of *Afzelia africana* and *Detarium microcarpum* seed oil. *Bollettino Chimico Farmaceutico.* *138*, 165-168.

Nkafamiya, I. I., Osemeahon, S. A., Dahiru, D., & Umaru, H. A. (2007). Studies on the chemical composition and physicochemical properties of the seeds of baobab (*Adasonia digitata*). *African Journal of Biotechnology,* *6*, 756-759.

Nnam, N. M., & Obiakor, P. N. (2003). Effect of fermentation on the nutrient and antinutrient composition of baobab (*Adansonia digitata*) seeds and rice (*Oryza sativa*) grains. *Ecology of Food and Nutrition,* *42*, 265-277.

Nordeide, M. B., Harløy, A., Følling, M., Lied, E., & Oshaug, A. (1996). Nutrient composition and nutritional importance of green leaves and wild food resources in an agricultural district, Koutiala, in Southern Mali. *International Journal of Food Sciences and Nutrition,* *47*, 455-468. https://doi.org/10.3109/09637489609031874

Nyanga, K. L., Gadaga, H. T., Nout, J. R. M., Smid, J. E., Boekhout, T., & Zwietering, H. M. (2013). Nutritive value of masau (*Ziziphus mauritiana*) fruits from Zambezi Valley in Zimbabwe. *Food Chemistry,* *138*, 168-172. https://doi.org/10.1016/j.foodchem.2012.10.016

Offor, C. E., Onwe, N. J., Agbafor, K. N., & Nwangwu, S. C. (2014). Determination of proximate and vitamin compositions of *Blighia unijugata* leaves. *Academic Journal of Nutrition*, *3*, 22-25. DOI: 10.5829/idosi.ajn.2014.3.3.9429

Ogunlade, I., Ilugbiyin, A., & Ajayi, I. O. (2011). A comparative study of proximate composition, antinutrient composition and functional properties of *Pachira glabra* and *Afzelia africana* seed flours. *African Journal of Food Science,* *5*, 32-35.

Ogunwa, T. H., Fasimoye, R. Y., Sholanke, D. R., Ademoye, T. A., Ilesanmi, O. C., Awe, O. B., Oloye, O. B., & Ajiboye, S. A. (2016). Compositional studies of *Baphia pubescens* (urohun) leaves. *Asian Journal of Natural & Applied Sciences,* *5*, 53-62.

Ogunyinka, B. I., Oyinloye, B. E., Osunsanmi, F. O., Kappo, A. P., & Opoku, A. R. (2017). Comparative study on proximate, functional, mineral, and antinutrient composition of fermented, defatted, and protein isolate of *Parkia biglobosa* seed. *Food science & nutrition,* *5*, 139-147. doi: 10.1002/fsn3.373

Ojeler, E, O. O. (2014). Phytochemicals, proximate, mineral element composition and antimicrobial activity of some selected medicinal plant seeds. Master of Science (M.Sc) in industrial chemistry, University of Ibadan, Ibadan, Nigeria.

Okullo, J. B. L., Omujal, F., Agea, J. G., Vuzi, P. C., Namutebi, A., Okello J. B. A., & Nyanzi, S. A. (2010). Proximate and mineral composition of shea (*Vitellaria paradoxa* C.F. Gaertn) fruit pulp in Uganda. *African Journal of Food, Agriculture, Nutrition and Development,* *10*, 4430-4443.

Okwu, D. E., & Morah, F. N. I. (2004). Mineral and nutritive value of *Dennettia tripetala* fruits. *Fruit,* *59*, 437-442. https://doi.org/10.1051/fruits:2005006

Okwu, D. E., & Nnamdi, F.U. (2008). Evaluation of the chemical composition of *Dacryodes edulis* and *Raphia hookeri* mann and wendl exudates used in herbal medicine in south eastern Nigeria. *African Journal of Traditional, Complementary and Alternative Medicines,* *5*, 194-200.

Oladejo, T. A. (2009). Proximate composition and micronutrient potentials of three locally available wild fruits in Nigeria. *African Journal of Agricultural Research,* *4*, 887-892.

Olujobi, O. J. (2015). Evaluation of the nutritive composition of five indigenous tree leaves used as vegetable in Ekiti State. *Journal of Agriculture and Environmental Sciences,* *4*, 185-197. http://dx.doi.org/10.15640/jaes.v4n1a23

Omale, J., Adeyemi, A. R., & Omajali, B. J. B. (2010). Phytoconstituents, proximate and nutrient investigations of *Saba florida* (Benth.) from Ibaji forest. *International Journal of Nutrition and Metabolism,* *2*, 88-92.

Onweluzo, J. C., & Morakinyo, A. O. (1997). Effect of pre-dehulling treatments on the composition of seeds of the legume *Afzelia africana* and its potential use in pastries. *Plant Foods for Human Nutrition (Formerly Qualitas Plantarum),* *50*, 203-210.

Osabor, V. N., Etiuma, R. A., & Ntinya, M. U. (2016). Chemical profile of leaves and roots of miracle fruit (*Synsepalum dulcificum*). *American Chemical Science Journal,* *12*, 1-8. DOI: 10.9734/ACSJ/2016/20456

Osman, M. A. (2004). Chemical and nutrient analysis of baobab (*Adansonia digitata*) fruit and seed protein solubility. *Plant Foods for Human Nutrition (Formerly Qualitas Plantarum),* *59*, 29-33.

Otitoju, G. T. O., Nwamarah, J. U., Otitoju, O., & Iyeghe, L. U. (2014). Nutrient composition of some lesser known green leafy vegetables in Nsukka Lga of Enugu State. *Journal of Biodiversity and Environmental Sciences,* *4*, 233-239.

Otori, A. A., & Mann, A. (2014). Determination of chemical composition, minerals and anti-nutritional factors of two wild seeds from Nupeland, North central Nigeria. *American Journal of Chemistry and Application,* *1*, 20-26.

Parkouda, C., Sanou, H., Tougiani, A., Korbo, A., Nielsen, D. S., Tano-Debrah, K., & Jensen, J. S. (2012). Variability of baobab (*Adansonia digitata* L.) fruits’ physical characteristics and nutrient content in the West African Sahel. *Agroforestry Systems,* 85, 455-463. https://doi.org/10.1007/s10457-011-9406-3

Pugalenthi, M., Vadivel, V., Gurumoorthi, P., & Janardhanan, K. (2004). Comparative nutritional evaluation of little known legumes, *Tamarindus indica*, *Erythrina indica* and *Sesbania bispinosa*. *Tropical and Subtropical Agroecosystems,* *4*, 107-123.

Raimi, M. M., Oyekanmi, A. M., & Farombi, A. G. (2014). Proximate and phytochemical composition of leaves of *Ceiba pentandra*, *Manihot esculentus* and *Abelmoschus esculentus* in Southwestern Nigeria. *Scientific Research Journal,* *2*, 30-34.

Saka, J. D. K., & Msonthi, J. D. (1994). Nutritional value of edible fruits of indigenous wild trees in Malawi. *Forest Ecology and Management,* *64*, 245-248. https://doi.org/10.1016/0378-1127(94)90298-4

Savadogo, A., Ilboudo, A. J., & Traoré, A. S. (2011). Nutritional potentials of *Acacia macrostachya* (Reichend) ex Dc seeds of Burkina Faso: Determination of chemical composition and functional properties. *Journal of Applied Sciences Research,* **7**, 1057-1062.

Siddhuraju, P., Vijayakumari, K., & Janardhanan, K. (1995). Nutritional and anti-nutritional properties of the underexploited legumes *Cassia laevigata* Willd. and *Tamarindus indica* L. *Journal of Food Composition and Analysis,* *8*, 351-162. https://doi.org/10.1006/jfca.1995.1030

Stadlmayr, B., Charrondiere, U. R., Eisenwagen, S., Jamnadass, R., & Kehlenbeck, K. (2013). Nutrient composition of selected indigenous fruits from sub-Saharan Africa. *Journal of the Science of Food and Agriculture,* *93*, 2627-2636. DOI 10.1002/jsfa.6196

Temitope, O. O., Fasusi, O. A., Ogunmodede, A. F., Thonda, A. O., Oladejo, B. O., Yusuf-Babatunde, A. M., & Ige, O. O. 2016. Phytochemical composition and antimicrobial activity of *Daniella oliveri* extracts on selected clinical microorganisms. *International Journal of Biochemistry Research & Review,* *14*, 1-13. DOI: 10.9734/IJBCRR/2016/28764

Ugese, F. D., Baiyeri, K. P., & Mbah, B. N. (2010). Proximate traits of the seed and seed cake of shea butter tree (*Vitellaria paradoxa* CF Gaertn.) in Nigeria’s savanna ecozone. *Journal of Applied Biosciences*, *31*, 1935-1941.

Ugese, F. D., Baiyeri, P. K., & Mbah, B. N. (2008). Nutritional composition of shea (*Vitellaria paradoxa*) fruit pulp across its major distribution zones in Nigeria. *Fruits,* *63*, 163-170. https://doi.org/10.1051/fruits:2008006

Usunobun, U., & Egharebva, E. (2014). Phytochemical analysis, proximate and mineral composition and in vitro antioxidant activities in *Telfairia occidentalis* aqueous leaf extract. *Journal of Basic and Applied Sciences,* *1*, 74-87.

Yisa, J., Egila, J. N., & Darlinton, A. O. (2010). Chemical composition of *Annona senegalensis* from Nupe land, Nigeria. *African Journal of Biotechnology,* *9*, 4106-4109.

Yusuf, A. A., Mofio, B. M., & Ahmed, A. B. (2007). Proximate and mineral composition of *Tamarindus indica* Linn 1753 Seeds. *Science World Journal,* *2*, 1-4

**Appendix legend**

Appendix 1. Average values of proximate composition in seeds

Appendix 2. Average values of proximate composition in fruit parts (apart from the seed)

Appendix 3. Average values of proximate composition in leaves

Appendix 4. Species abbreviations
